# Adult Epstein–Barr virus encephalitis: a retrospective case report series with metagenomic next-generation sequencing-based diagnosis

**DOI:** 10.3389/fneur.2026.1861146

**Published:** 2026-09-16

**Authors:** Yinghui Li, Zhejun Yu, Meili Shen, Shaobo Cui, Xinjing Yang

**Affiliations:** 1 Department of Emergency, Honghui Hospital, Xi'an Jiaotong University, Xi'an, China; 2 Department of Intensive Care Unit, The First Affiliated Hospital of Soochow University, Suzhou, China; 3 Department of Medecine, Dinfectome Inc., Nanjing, China; 4 Department of Critical Care Medicine, Jincheng General Hospital, Jincheng, China

**Keywords:** clinical features, encephalitis, Epstein–Barr virus, metagenomic next-generation sequencing, prognosis, treatment

## Abstract

**Background:**

EBV encephalitis is uncommon in adults, and its diagnosis and management remain challenging, particularly in patients with underlying medical conditions.

**Methods:**

We retrospectively analyzed seven adult patients with EBV encephalitis confirmed through cerebrospinal fluid (CSF) metagenomic next-generation sequencing (mNGS) between December 2021 and January 2024.

**Results:**

Six patients (86%) were female, with a median age of 59 years. Five patients had significant underlying conditions, including malignancies (*n* = 2), systemic lupus erythematosus (*n* = 1), and EBV-associated hemophagocytic lymphohistiocytosis (HLH, *n* = 2). Common clinical presentations included fever (86%), impaired consciousness (86%), neck stiffness (57%), headache (43%), and decreased muscle strength (43%). CSF analysis revealed elevated protein levels (57%) and leukocytosis (71%). Magnetic resonance imaging (MRI) identified encephalitis-associated lesions in three of six patients (50%), whereas computed tomography (CT) was largely unremarkable. All patients received antiviral therapy, and five additionally received corticosteroids. Three patients recovered, whereas four died, with all deaths occurring in patients with concurrent malignancies or HLH.

**Conclusion:**

MRI is the preferred imaging modality for suspected encephalitis, and CSF-mNGS is an essential pathogen detection assay. Underlying conditions such as malignancies and HLH are associated with poor clinical outcomes.

## 1 Introduction

Epstein–Barr virus (EBV), also known as *human herpesvirus 4*, infects nearly 95% of adults worldwide and is transmitted primarily through saliva. Transmission can also occur through the clinical transplantation of EBV-positive organs (1). EBV employs multiple strategies for immune evasion, thereby maintaining viral latency and allowing the virus to persist in the host throughout the host’s lifetime. Primary EBV infection is often asymptomatic; however, reactivation of the virus can lead to a wide spectrum of illnesses (2, 3). Infectious mononucleosis is a common clinical manifestation of EBV infections. In addition, EBV has been associated with various autoimmune diseases, including systemic lupus erythematosus (SLE) and multiple sclerosis (4). EBV was the first virus to be identified as a causative agent of human tumors, including Burkitt’s lymphoma, nasopharyngeal carcinoma, Hodgkin’s lymphoma, non-Hodgkin’s lymphoma, T-cell lymphoma, and NK/T-cell lymphoma (5).

EBV can compromise the integrity of the blood–brain barrier, potentially leading to encephalitis when it infects neurons (6). EBV encephalitis is relatively uncommon. In a prospective cohort study of encephalitis, EBV was the seventh most common cause of infectious encephalitis and the third most common cause of Herpesviridae encephalitis (7). The clinical features of EBV encephalitis are similar to those of other viral encephalitis and are non-specific (8). The principal symptoms are fever, headache, and impaired consciousness. Magnetic resonance imaging (MRI) is a non-specific test, and only a few patients exhibit abnormalities on MRI (9). Therefore, it is challenging to confirm the diagnosis of EBV encephalitis based on clinical features and imaging findings; pathogen testing is essential for accurate diagnosis.

Polymerase chain reaction (PCR) is a commonly used method for detecting EBV in the cerebrospinal fluid (CSF); however, empirical antiviral therapy is frequently administered to patients with encephalitis, which can influence the accuracy of viral detection. A low viral load may also be a contributing factor (10). The advent of genetic diagnostic technologies has led to greater utilization of next-generation sequencing technology in the diagnosis of encephalitis, and metagenomic next-generation sequencing (mNGS) is more accurate than PCR and can significantly improve diagnostic rates (11). The treatment of EBV encephalitis is primarily based on antiviral agents, such as ganciclovir and acyclovir, with corticosteroids and immunoglobulins sometimes used in combination. However, there is still a lack of consensus regarding specific treatment strategies (12, 13). Given the rarity of EBV encephalitis and the uncertainties surrounding its diagnosis and treatment, we present a case report series of seven adult patients with EBV encephalitis who were admitted to our hospital over the past 2 years, with the aim of raising awareness of the disease.

## 2 Materials and methods

### 2.1 Patient enrollment

This study was a retrospective case report series that included patients diagnosed with EBV encephalitis who were treated at the First Affiliated Hospital of Soochow University between December 2021 and January 2024. During the study period, a total of 231 adult patients underwent CSF mNGS testing for suspected or screened central nervous system (CNS) infections. Among them, 16 patients tested positive for EBV by CSF-mNGS, and 7 patients were ultimately diagnosed with EBV encephalitis. All patients with EBV encephalitis in this study were diagnosed based on clinical and etiological criteria. The clinical criteria for encephalitis included: (1) fever, altered mental status, and abnormal cerebrospinal fluid (CSF) cytology; (2) brain parenchymal lesions on neuroimaging; or (3) abnormal electroencephalogram (EEG) findings. The etiological criteria included: (1) detection of EBV nucleic acid in CSF by PCR or mNGS; or (2) detection of EBV-specific immunoglobulin M (IgM) antibodies in CSF and/or serum.

### 2.2 Data collection and analysis

Clinical data were collected from seven patients with EBV encephalitis. The electronic and paper-based medical records of each patient, including general information, clinical features, laboratory findings, imaging manifestations, treatment, and prognosis, were reviewed, and a clinical database was created for analysis. The prognoses of the seven patients were followed up via telephone.

## 3 Results

### 3.1 General information

Clinical characteristics of patients with EBV encephalitis are summarized in Table 1. Of the seven patients diagnosed with EBV encephalitis, six were female (86%). The median age of the patients was 59 years (range, 27–69 years). None of the patients had a history of EBV infection. Two patients (cases 4 and 1) had a history of malignancy, including extranodal NK/T-cell lymphoma (nasal type) and a malignant tumor of the maxillary sinus. The first patient received regular postoperative radiotherapy, whereas the second patient received postoperative oral methylprednisolone. A patient (case 6), who had been diagnosed with SLE for more than 10 years and was routinely treated with oral prednisone and hydroxychloroquine, exhibited a negative test result for SLE during hospitalization. Patients (cases 2 and 5) were in good health in the past but were diagnosed with EBV-associated HLH during hospitalization. Patient 5 underwent hematopoietic stem cell transplantation (HSCT). Before transplantation, the copy number of blood EBV DNA was 2.55×10^3^. In contrast, patient 2 did not undergo transplantation.

**Table 1:** General information of patients with EBV encephalitis.

Patients/cases	Gender	Age (yrs)	Medical history	Principal diagnosis on admission	Main clinical manifestations	Treatment administered before MRI/CT	Timing of first MRI/CT after symptom onset (days)	Electroencephalography (EEG)	MRI	CT	Treatment after diagnosis	Hospitalization (days)	Outcome
1	Male	69	Malignant tumor of maxillary sinus (postoperative) and oral methylprednisolone	Sepsis, EBV encephalitis	Fever, impaired consciousness, malaise, and epilepsy	Foscarnet sodium	24/19	NA	Abnormal	Normal	Foscarnet sodium	25	Death
2	Female	44	None	EBV-associated HLH and EBV encephalitis	Fever, impaired consciousness, stiff neck, sore throat, coughing, and malaise	Foscarnet sodium	5/2	NA	Normal	Normal	Foscarnet sodium	13	Death
3	Female	47	None	EBV encephalitis	Fever, impaired consciousness, stiff neck, and olfactory dysfunction	Foscarnet sodium	3/1	Normal	Abnormal	Normal	Acyclovir and methylprednisolone	35	Recovery and memory loss
4	Female	60	Extranodal NK/T-cell lymphoma, nasal type (postoperative), and postoperative radiotherapy	EBV encephalitis	Headache, numbness of the limbs, decreased muscle strength, and nociceptive sensitization	Ganciclovir, methylprednisolone, and gamma globulin	7/NA	NA	Normal	NA	Ganciclovir, methylprednisolone, and gamma globulin	18	Death
5	Female	27	None	EBV-associated HLH, EBV encephalitis	Fever, impaired consciousness, coughing, and epilepsy	Ganciclovir, methylprednisolone, and gamma globulin	NA/3	Abnormal	NA	Normal	Ganciclovir, methylprednisolone, and gamma globulin	61	Death
6	Female	59	SLE over 10 years, oral prednisone and hydroxychloroquine	EBV encephalitis	Fever, impaired consciousness, stiff neck, headache, gastrointestinal symptoms, decreased muscle strength, coughing, and limb tremor	Foscarnet sodium and methylprednisolone	8/4	NA	Abnormal	Normal	Ganciclovir, methylprednisolone, and gamma globulin	13	Recovery
7	Female	66	None	EBV encephalitis	Fever, impaired consciousness, stiff neck, headache, sore throats, gastrointestinal symptoms, decreased muscle strength, and malaise	Foscarnet sodium	10/3	NA	Normal	Normal	Ganciclovir, methylprednisolone, and gamma globulin	32	Recovery

The case numbers were sorted according to the time of admission. EBV, Epstein–Barr virus; HLH, hemophagocytic lymphohistiocytosis; SLE, systemic lupus erythematosus; MRI, magnetic resonance imaging; CT, computed tomography; NA, not available.

### 3.2 Clinical features

Clinical features of EBV encephalitis can be classified into two categories: non-neurological and neurological features (Table 1). In terms of non-neurological features, six patients (6/7, 86%) exhibited fever accompanied by malaise and coughing. Two patients (2/7, 29%) exhibited notable gastrointestinal symptoms, and two patients (2/7, 29%) reported sore throats. Only one patient (1/7, 14%) presented with numbness of the limbs as the primary symptom. The most common neurological features were impaired consciousness (6/7, 86%), stiff neck (4/7, 57%), headache (3/7, 43%), and decreased muscle strength (3/7, 43%). Olfactory dysfunction, nociceptive sensitization, limb tremor, and epilepsy were observed in no more than three cases. Impaired consciousness manifested as inappropriate responses, apathy, and varying degrees of coma.

### 3.3 Laboratory findings

The etiological diagnosis of EBV encephalitis in this study was based on the results of CSF mNGS, with the number of EBV-specific sequence reads ranging from 3 to 36,018 (Table 2). Samples with ≥3 specific reads were considered positive for EBV. Using clinical diagnosis as the gold standard, we calculated the diagnostic performance indicators of CSF mNGS for detecting EBV encephalitis. The sensitivity, specificity, positive predictive value (PPV), negative predictive value (NPV), and accuracy were 100, 95.98, 43.75, 100, and 96.10%, respectively (Table S1). Furthermore, *human herpesvirus type 1* was identified in the CSF of case 3, with 133sequence reads. No other viruses were detected in the CSF samples from the remaining patients.

**Table 2:** Laboratory findings in patients with EBV encephalitis.

Patients/cases	Timing of CSF examination after symptom onset (days)	EBV reads in CSF (mNGS)	CSF oligoclonal bands (OCB)	EBV copies^*^ or reads in blood (PCR^*^ or mNGS)	CSF protein (0.15–0.45 g/L)	CSF glucose (2.5–4.5 mmol/L)	CSF chloride (120—130 mmol/L)	CSF leukocytes (×10^6^/L)	CSF monocyte (%)	CSF pathology
1	1	3	NA	NA	4.09↑	3.84	113↓	58↑	62.1	NA
2	6	5	NA	3.19 × 10^3*^	0.41	6.61↑	143.5↑	3	0	NA
3	3	33	Negative	NA	0.2	4.08	105.3↓	44↑	97.7	A large number of lymphocytes
4	1	7,503	Negative	3.12 × 10^4*^	2.58↑	1.41↓	102.4↓	77↑	93.5	Few lymphocytes
5	0	185	NA	<1 × 10^2*^	0.45	3.99	125.1	3	33.3	NA
6	3	36,018	NA	4	0.492↑	2.98	140.4↑	71↑	95.9	NA
7	6	4	NA	<1 × 10^2*^	1.15↑	1.98↓	112.7↓	107↑	67.3	Normal

The timing of blood EBV detection was essentially concurrent with that of the lumbar puncture and CSF EBV detection. EBV, Epstein–Barr virus; CSF, cerebrospinal fluid; mNGS, metagenomic next-generation sequencing; the results were expressed as the number of reads; PCR^*^, polymerase chain reaction; the results were presented in copies per milliliter (copies/mL); NA, not available.

Routine CSF and biochemical findings in patients with EBV encephalitis were not specific (Table 2). CSF pressure was within the normal range in four patients, decreased in one patient, increased in one patient, and was unrecorded in one patient. The CSF was colorless and clear in all cases. Four patients exhibited elevated CSF protein levels, with the highest level reaching 4.09 g/L. CSF glucose levels were within the normal range in four patients, decreased in two patients, and increased in one case; CSF chloride levels were decreased in four patients, increased in two patients, and were normal in one patient; and CSF leukocyte counts were elevated in five patients, fluctuating between 44 and 107 × 10^6^/L. In addition, a high percentage of monocytes was observed. CSF pathology was also documented in three cases, with no instances of heterogeneous cells observed.

The presence of EBV in the blood was also used as an indicator. Blood samples from four patients were tested for EBV using PCR. Two patients tested positive for EBV, whereas the remaining two tested negative. In a separate case, EBV was identified by blood mNGS, with a sequence read count of 4.

### 3.4 Imaging manifestations

Electroencephalography (EEG) was documented in two of the seven cases. In one case, the EEG showed a severe background abnormality characterized by periodic compound waves in the left hemisphere. This patient had documented seizures. The EEG of the other patient showed no abnormalities. Cranial computed tomography (CT) revealed no significant abnormalities in six patients.

Cranial MRI scans were documented in six patients, and findings suggestive of encephalitis were observed in three cases (Figure 1). Lesions were identified in several areas of the brain, including the centrum semiovale, frontoparietal junction, temporo-occipital lobe, parietal anterior horn of the lateral ventricle, pontine arm, insula, hippocampus, cerebellar hemisphere, frontal lobe, and parieto-occipital lobe. Lesions of the temporal lobe, insula, and centrum semiovale were noted in two patients.

**Figure 1:**
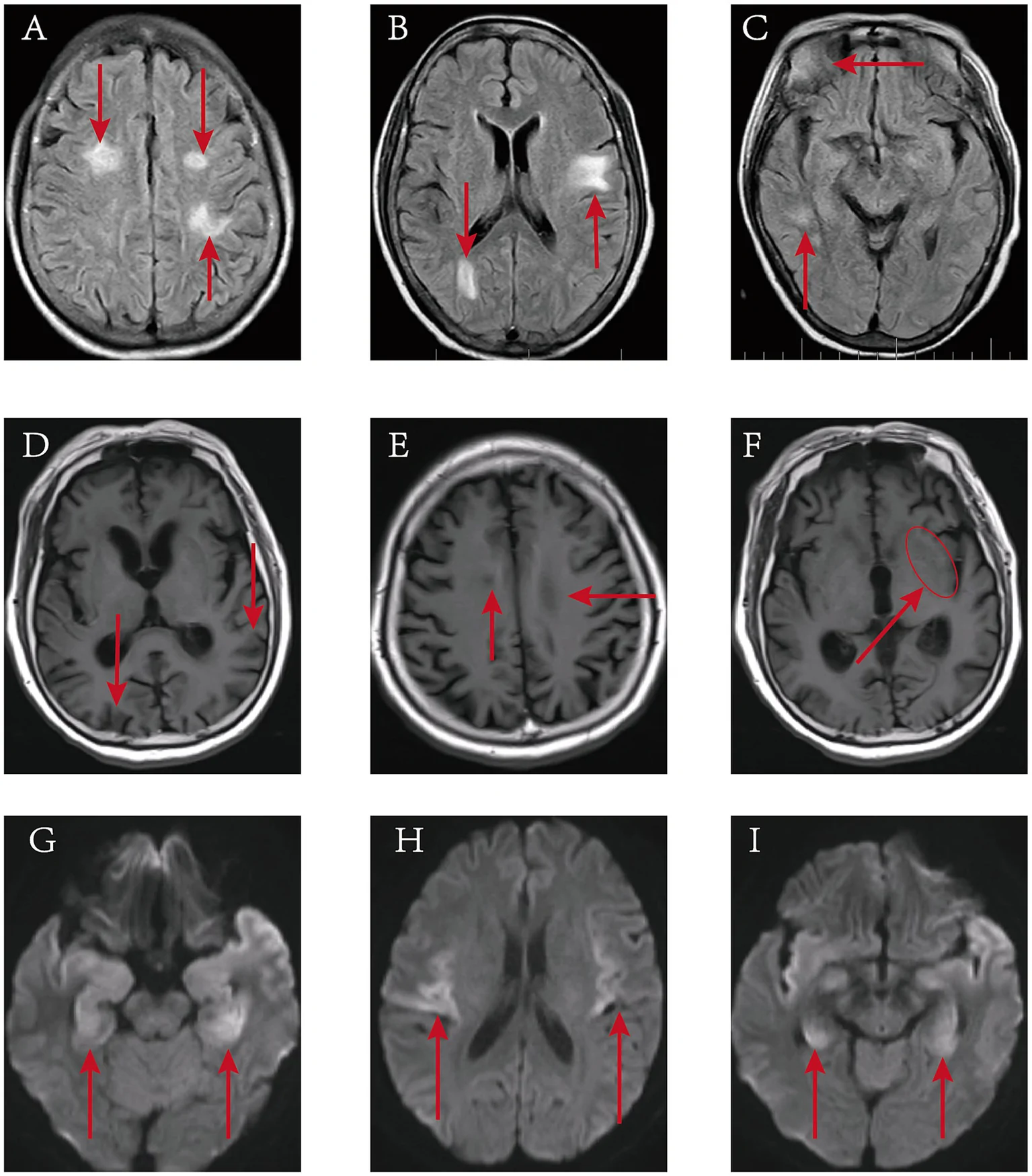
Representative MRI images of three cases with abnormal findings. Each row represents one case, corresponding to cases 6, 1, and 3, respectively. **(A–C)** show abnormal signal intensities in the bilateral frontal lobes, left temporal lobe, and right parieto-occipital lobe of case 6; **(D–F)** demonstrate abnormal signal intensities in the bilateral temporo-occipital lobes, periventricular regions, and left insula of case 1; **(G–I)** reveal swelling with signal alterations involving the bilateral temporo-insular regions and bilateral hippocampi in case 3. Red arrows indicate the locations of the lesions. MRI, magnetic resonance imaging.

### 3.5 Treatment and prognosis

Case 1, who had previously been diagnosed with a malignant tumor of the maxillary sinus, and case 2, who had been diagnosed with EBV-associated HLH during hospitalization, were treated with foscarnet sodium as antiviral therapy. Despite the confirmed diagnosis of EBV encephalitis, these two patients did not undergo antiviral therapy adjustment or receive corticosteroids or gamma globulins and ultimately died. Four patients (cases 4, 5, 6, and 7) were treated with ganciclovir and intravenous methylprednisolone and gamma globulin. Of these patients, case 4, who had previously been diagnosed with extranodal NK/T-cell lymphoma (nasal type), and case 5, who had been diagnosed with EBV-associated HLH during hospitalization, exhibited more rapid progression and ultimately died. The remaining two patients were discharged with an improvement in their condition and no apparent residual effects. Patient 3 was treated with acyclovir and intravenous methylprednisolone but not with gamma globulins. She had a favorable prognosis. She subsequently returned to the hospital because of hallucinations and memory loss. She was discharged after receiving comprehensive treatment; however, she continued to experience residual anxiety and memory loss.

## 4 Discussion

EBV encephalitis is relatively uncommon, accounting for 5–18% of acute viral encephalitis cases. The prognosis is favorable in the majority of patients, with only a small proportion developing severe neurological complications or dying (14). Moreover, EBV positivity in CSF was not independently associated with unfavorable outcomes among patients with suspected intracranial infection (15). The clinical manifestations of EBV encephalitis are non-specific, and the majority of patients exhibit only mild neurological symptoms; consequently, neurological damage caused by EBV infection is often overlooked by clinicians. To date, only a few single-center or retrospective studies have been conducted in pediatric patients (16, 17).

In a review of 104 cases of EBV encephalitis reported worldwide over the past 25 years, Peuchmaur et al. found that 58% of the patients were male, the median age was 22 years, 20% were immunocompromised, and the mortality rate was 16% (18). Another retrospective study demonstrated that of the 48 cases of EBV encephalitis occurring in individuals over 40 years of age, 37 (77.1%) were female, and the mortality rate was 0%. No underlying diseases or immune status were reported (17). The cases in our study consisted of six female patients (86%), with a median age of 59 years. The majority of patients had underlying diseases, and the mortality rate was high (57%).

The clinical presentation of EBV encephalitis is not specific, with patients typically experiencing fever and neurological symptoms, including impaired consciousness, delirium, and hallucinations. Neck stiffness is less common (18) but was present in four of the seven cases in our study. In patients with EBV encephalitis, CSF is typically characterized by elevated leukocyte counts and protein levels (15, 18). Similarly, our study found elevated leukocyte counts in five (71%) patients and elevated protein levels in four (57%) patients. Peuchmaur et al. observed that MRI revealed encephalitis-associated lesions in 69% of cases, mostly involving the cerebellum, basal ganglia, and frontal lobes (18). These findings suggest that MRI is an effective imaging modality. Cranial MRI was documented in six patients in our study and was suggestive of encephalitic lesions in three (50%). In contrast, CT had limited diagnostic value.

PCR has historically been a valuable tool for confirming the diagnosis of EBV encephalitis (10). Anti-EBV IgM also plays an important role as a supplementary diagnostic test (18). Recent studies have also used CSF mNGS for pathogen detection (19). PCR is a targeted detection method with advantages including a well-established workflow, relatively high specificity, and the ability to quantify viral load. mNGS can detect multiple pathogens simultaneously and is advantageous for encephalitis of unknown etiology or for the differential diagnosis of mixed infections (20, 21). However, mNGS results may be affected by sample quality, pathogen load, reference databases, and contamination, and their diagnostic value also depends on clinical relevance. Therefore, neither PCR nor mNGS should be used independently of clinical manifestations and ancillary findings to establish a diagnosis of EBV encephalitis. All seven cases in this study tested positive for EBV by CSF mNGS, enabling accurate and rapid diagnosis. However, the detection of EBV by CSF mNGS is not equivalent to a definitive diagnosis of EBV encephalitis. EBV can persist lifelong in B lymphocytes, and viral reactivation may occur in patients with immune dysfunction, malignancy, or exposure to immunosuppressive therapies. Therefore, the detection of EBV-DNA or EBV sequences in CSF may reflect active viral replication within the central nervous system, but it may also be related to blood–brain barrier disruption, peripheral blood contamination, or the entry of B cells carrying latent EBV into the CSF. In addition, low-level or incidental detection of EBV does not necessarily indicate direct viral invasion of the brain parenchyma. Therefore, in this study, we did not consider CSF mNGS positivity as a standalone diagnostic criterion. Instead, the diagnosis was made by integrating encephalitis-related clinical manifestations, CSF inflammatory changes, brain MRI and/or EEG findings, results of testing for other pathogens, and serum and peripheral blood EBV test results. For patients with underlying malignancy, special attention should be paid to differentiating between EBV reactivation, tumor-related central nervous system involvement, immune-mediated encephalitis, and other opportunistic infections, thereby reducing the risk of misclassifying incidental EBV detection as EBV encephalitis. The specificity of mNGS (95.98%) further supports this conclusion: a positive EBV result by CSF-mNGS does not imply EBV encephalitis. In contrast, the 100% sensitivity further demonstrates the advantages of CSF-mNGS for diagnosing EBV encephalitis. Furthermore, five patients had blood EBV-DNA tested concurrently with lumbar puncture to evaluate systemic EBV activation status. Of note, isolated positive blood EBV-DNA cannot establish a diagnosis of EBV encephalitis; this finding indicates only systemic viremia or viral reactivation. In our study cohort, EBV was detected in the CSF of all seven patients, which constituted the primary etiological evidence for EBV encephalitis. In addition, discordance was observed between blood and CSF EBV results. Concurrent positivity in both blood and CSF may indicate systemic viremia combined with central nervous system (CNS) invasion. Some patients exhibited positive CSF EBV results but negative peripheral blood EBV results, suggesting that viral replication may be restricted to the CNS.

In terms of treatment, acyclovir and ganciclovir are commonly used, whereas corticosteroids and immunoglobulins are important adjuncts (18). Four patients in this study were treated with ganciclovir combined with methylprednisolone and gamma globulin.

The regression of EBV encephalitis is closely associated with the medical history of a patient. EBV is associated with numerous malignant tumors and represents a global burden (22, 23). Two of the 104 cases of EBV encephalitis reported by Peuchmaur et al. (18) involved malignancies, specifically breast and lung cancers. These cases highlight the fact that the patients were immunosuppressed following chemotherapy, rather than that the cancers themselves were associated with EBV infection. To our knowledge, this is the first report of two cases of EBV encephalitis in patients previously diagnosed with maxillary sinus malignancy and extranodal NK/T-cell lymphoma, both of which were associated with EBV. Two patients were treated with antiviral therapy, but ultimately died. In the first case, orbital infiltration of the malignant tumor was diagnosed during hospitalization. In the second case, intracranial infiltration could not be ruled out. Therefore, the deaths of these two patients may have been associated with progression of their malignant tumors. EBV infection is also a significant factor in HLH (24). Patients with EBV-associated HLH who present with mild encephalitis have a favorable prognosis following treatment; however, the outcomes of patients with more severe disease remain unknown (25). Case 5, in addition to recurrent EBV infections, exhibited mutations in the HLH‑related genes IL-2-inducible T-cell kinase (ITK) and Unc‑13 homolog D (UNC13D) (+). Since the diagnosis of primary HLH was considered, the patient underwent HSCT while EBV DNA was positive in the blood at the time of transplantation and was in a state of myelosuppression following transplantation. On the other hand, case 2 did not undergo HSCT. Following the diagnosis, both patients received different antiviral regimens, but both ultimately died. We identified a patient with EBV encephalitis and a history of SLE whose SLE disease activity was assessed during hospitalization and was negative. The patient was treated and discharged after clinical improvement. These cases indicate that patients with tumors, autoimmune diseases, or HLH should undergo CSF examinations as soon as possible to evaluate for EBV encephalitis when presenting with fever and neurological symptoms.

This study had some limitations. First, the limited sample size precluded a comprehensive assessment of the efficacy of different treatment options. Second, the retrospective design may introduce selection bias and result in incomplete data. Further well-designed studies are warranted to overcome these limitations. In addition, the potential influence of malignant tumor progression cannot be ruled out.

## 5 Conclusion

This study demonstrated the crucial role of CSF mNGS and cranial MRI in the diagnosis of EBV encephalitis. The majority of EBV encephalitis cases in this study were associated with underlying diseases, including SLE, malignancy, and HLH. These patients exhibited high morbidity and mortality rates, suggesting that the clinical outcomes were closely associated with the underlying conditions of the patients. These cases provide clinical experience and serve as important considerations for the clinical diagnosis and treatment of EBV encephalitis.

## Data Availability

The original contributions presented in the study are included in the article/Supplementary material, further inquiries can be directed to the corresponding author/s.
