# Supplementary material for: Adult Epstein–Barr virus encephalitis: a retrospective case report series with metagenomic next-generation sequencing-based diagnosis
Source: Front Neurol. 2026 Sep 16;17:1861146. doi: 10.3389/fneur.2026.1861146 (PMC13624100; doi:10.3389/fneur.2026.1861146)
Supplement: Supplementary file 1 [file Table_1.DOCX]

|  |  | EBV encephalitis, n=7 | None-EBV encephalitis, n=224 | Sensitivity,% | Specificity,% | Positive predictive value (PPV), % | Negative predictive value (NPV), % | Accuracy, % |
| --- | --- | --- | --- | --- | --- | --- | --- | --- |
| CSF-mNGS | EBV + | 7 | 9 | 100% | 95.98% | 43.75% | 100% | 96.10% |
|  | EBV- | 0 | 215 |  |  |  |  |  |

TableS1 Performance results of CSF mNGS in the diagnosis of Epstein-Barr Virus encephalitis.

Note: CSF: cerebrospinal fluid, mNGS: metagenomic next-generation sequencing; EBV: Epstein-Barr Virus
